# Everybody's Page

**Published:** 1892-01-30

**Authors:** 


### Everybody's Page.

Los Angeles must be a very healthy place, and the Sisters' Orphan Asylum, situated there, must be very well managed. It is asserted that during the last three years there has been only one death in the asylum, which contains an average of two hundred and fifty children, mostly under four years of age.

In concluding our report of San Francisco, we wish it to be understood that we excepted from our remarks the Hospital for Women and Children therein referred to, and also the small Military Hospital at Fort Mason under the charge of Dr. Perley. We deal this week with the Fort Mason Hospital, and though we had not time to visit that for women and children, we have it on reliable authority that it is well managed, and that the ladies of San Francisco have set the millionaires an example in good works, which the latter would do well to speedily follow.

The legislature of Michigan, if the *Annales d'Hygiene* is correctly informed, has taken stringent measures against cigarette smoking. It is now a misdemeanour punishable by fine to manufacture or sell cigarettes or cigarette papers in that State. Michigan would not be a bad place to send, for a lengthened period of residence, some of our gilded youths who are never without a cigarette in their mouths. A lesson in self-control and self-denial would be as beneficial to their morals, as a temporary cessation, which might be followed by moderation, would improve their physique. If there was more moderation in the use of tobacco we should hear of fewer cases of "irritable heart."

If bacilli are in the air there seems no reason why they should not be in the dust; in fact, a Viennese doctor, by his experience with some grapes which he bought, has been led to the unpleasant suggestion that the tubercle bacilli may be in the dust of our streets. After rinsing the dust from these grapes in pure spring water, he found the water very dirty. As an experiment, he injected some of this water into three guinea-pigs. One died in two days of peritonitis; the other two also died, but after a lapse of over a month. On examination the bodies showed pronounced tuberculosis originating in the site of inoculation. This is really most cheerful news, as if we are ever to rid ourselves of tuberculosis we must get rid of our dust. Here is an opening for enterprising gentlemen of an ingenious turn of mind.

Dr. Koch is said to believe firmly in the ultimate success of his cure. If he does relieve mankind from one of its numerous ills, the human race will not quarrel with him for his enthusiasm. His new publication will no doubt be expressive of his confidence that tuberculin will be quite free from any dangerous element after isolation of the active principle. Like many enthusiasts the doctor, no doubt, erred in overstating his case at first. Other eminent men have similarly failed. The late Bishop Blomfield, when a curate, preached a carefully prepared sermon which he congratulated himself would convince his hearers in a belief in the Trinity. After church one of his congregation, a farmer, walked home with him and expressed his admiration at the sermon; "but," said he, "Mr. Blomfield, notwithstanding *all you said to the contrary*, I still believe in the Trinity!"

